# Lipids productivity of cyanobacterium *Anabaena vaginicola* in an internally illuminated photobioreactor using LED bar lights

**DOI:** 10.1038/s41598-024-54414-0

**Published:** 2024-03-21

**Authors:** Hootan Goldoost, Farzaneh Vahabzadeh, Narges Fallah

**Affiliations:** Department of Chemical Engineering, Amirkabir University of Technology, No. 350, Hafez Street, Tehran, 1591634311 Iran

**Keywords:** Internally illuminated photobioreactor, Led light bar, Cyanobacterium *anabaena vaginicola*, Biomass formation, Lipids production, Chemical engineering, Environmental biotechnology, Plant biotechnology, Biofuels, Light responses, Plant biotechnology, Plant stress responses

## Abstract

Concerns over environmental issues exists and desire to decrease of their extent, have directed efforts toward green energy production. Growth behavior of *Anabaena vaginicola* was determined in a photobioreator which illuminated internally (IIPBR) using LED bar light. Excessive heat generated in the IIPBR was taken care of by applying a novel air-cooled system. Further note in experimentation was to find favorable cultivation conditions in the IIPBR for *A. vaginicola* growth and its lipids production capacity. The following results are expressed: 80 µmol photons m^−2^ s^−1^ as light intensity, 0.5 g/l as NaNO_3_, and 120 ml/min as CO_2_ amount being expressed in terms of aeration rate. The findings were interpreted in terms of a two-component system where the genes encoded to the relevant proteins are present in cyanobacteria and their expressiveness depends on environmental stress. By determining growth rate constant as 0.11 d^−1^, the productivity in terms of biomass formation was calculated as 202.6 mg L^−1^ d^−1^. While rate of lipids production by the test cyanobacterium is 15.65 mg L^−1^ d^−1^. Based on total energy used for IIPBR performance, biomass productivity per unit power input equals to 0.74 g W^−1^ d^−1^ and this is in favorable position compared with other photobioreactors.

## Introduction

Continuous decrease of fossil fuel sources, increase of population globally, and change in living standards worldwide all exert tension searching for a new source of energy. Significant efforts in recent years have been directed toward the potential of cyanobacteria and microalgae as energy sources where cultivation of these microorganisms in photobioreactors (PBRs) and obtainable biomass have received much attention^1^.

Arrangement of environmental factors for cultivation of cyanobacteria, as well-classified prokaryote, is that design of PBR provides nutritive condition in which the cells express their ability in controlling coexistence of nitrogen fixation and photosynthesis. Synthesis of nitrogenase as oxygen-sensitive enzyme and O_2_-producing photosynthetic process, both are needed to be accomplished accurately by the cells. i.e., production of ATP and NADPH in photosynthesis are required for synthesis of nitrogenase which catalytically is responsible for cellular fixation of N_2_^2^. Performance of cyanobacteria in nature considering its biological properties, is quite beneficiary. Contribution in improving soil functions has been well recognized and followings are some examples: increasing carbon and nitrogen contents of soil, having role in soil aggregation (a measure of soil ability in keeping its intact structure), stabilizing status of water holdup as capability of soil to keep its structural integrity^3^. Capability of cyanobacteria in performing oxygenic photosynthesis and fixation of atmospheric nitrogen simultaneously, has also given distinctive character to these prokaryotes in synthesizing variety of biomolecule with wide range of applications^4^. Developing innovative approaches in many cases is necessary. For instance, in clean technology subject when one focuses on bioenergy, the aim is to reduce extent of seriousness of environmental issues. Thus, production of biodiesel and biohydrogen is of interest using cyanobacteria, i.e., these biofuels have almost no roles in production of greenhouse gases^5–9^.

Importance of performing photosynthesis in an efficient manner, has given distinctive character to cyanobacteria: the cells sense environment and are able to change their physiological behavior and respond to fluctuation of carbon dioxide concentration by following a strategy which relies on CO_2_ concentration mechanism (CCM)^10^. The purpose of CCM is to support growth of cyanobacteria by letting the microorganism formulate accumulation of CO_2_ in the form of bicarbonate in carboxysomes and favorably respond to living under low level CO_2_ conditions. Carboxysomes as the proteinaceous micro-component contains ribulose biphosphate carboxylase/oxygenase (RuBisCO) and carbonic anhydrase where conversion of the absorbed bicarbonate to CO_2_ readily takes place and the operation is aimed to saturate activity of RuBisCO as the main enzyme in carboxylation. The complexity of RuBisCO also has been related to oxygenase activity of the enzyme by which O_2_ in presence of light takes place of CO_2_ as the substrate. Release of the fixed carbon through photorespiration is a burden for the cells in performing photosynthesis at efficient level. The value of half-saturation constant (K_s_) for RuBisCO in CO_2_ carboxylation shows that (in this serious competition between absorbing CO_2_ and O_2_) preference is for CO_2_ (Ksco_2_ = 20 µMolar and Kso_2_ = 200 µMolar)^1,2^.

In isolation of cyanobacteria from different rice cultivation provinces in Iran (perhumid and semi-arid regions), *Anabaena* and *Nostoc* have found to be the dominant genera^11^. Effects of several factors on *A. vaginicola* growth were studied in the present work: light (quantity and quality), CO_2_ supply in terms of the aeration, and nitrogen content of the culture medium. All these factors affect cell metabolism and biomass composition including lipids, carbohydrates, and proteins can be modulated in this manner^12^.

The reactor study of these influential factors is necessary before any scale up consideration on the biomass production. Based on using LED bar lights, an internally illuminated photobioreactor was designed (500 ml as the working capacity) to monitor growth behavior of *A. vaginicola* during its cultivation in BG11 medium. Variable parameters include light intensity (8, 80, and 150 µmol photons m^−2^ s^−1^), inorganic nitrogen content as NaNO_3_ (0, 0.5, and 1.5 g/l), and aeration expressed as CO_2_ content in the system (0, 40, and 120 ml/min). By defining a standard condition in terms of light intensity (80 µmol photons m^−2^ s^−1^), nitrogen content (1.5 g/l) and aeration rate (120 ml/min), the experiments were followed to monitor the response of the c. *Anabaena vaginicola* in a test system under stressful condition (8, and 150 µmol photons m^−2^ s^−1^), (0, and 0.5 g/l), (0, and 40 ml/min).

Further note in this study was to keep the photobioreactor system at a constant temperature, the effort was directed to position the LED light bars into a glass enclosure and contact of the bars with the aqueous medium was avoided. This configuration, with its two ports provided satisfactory conditions by letting the fresh air enter from one port and the warm air was forced to exit from another port (see the experimental section for the details).

As described above, the purpose is to identify the extent of lipid production of *A. vaginicola*, through its cultivation in the IIPBR designed in the present study. Light intensity, CO_2_ level, and inorganic nitrogen content of the growth medium as environmental factors will be undertaken by the test microorganism. By calculation of produced biomass and lipids, performance of the IIPBR will be evaluated and compared to other PBRs in refences. This will lead to finding a method for efficient and economical production of *A. vaginicola*.

**Figure 1:**
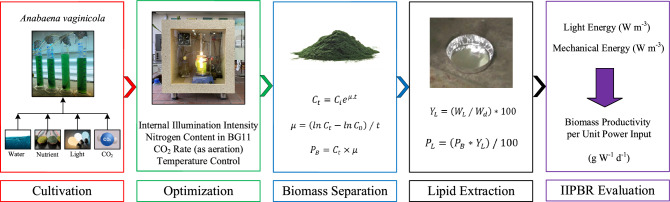
Sequence of the work in the present study: 1—A. vaginicola Cultivation, 2—Optimization in terms of light intensity, nitrogen content of the system, and CO_2_ content based on aeration rate while controlling the IIPBR temperature at constant level. 3—Biomass separation, 4—lipid extraction, and 5—Evaluation of the IIPBR performance.

The significance of this research is that the results are extendible by conducting a soil experiment by using grown *A. vaginicola* as soil inoculant to poor soils. It is noted that there is limited data in the literature for evaluation of *A. vaginicola* growth and assessment of key parameters play a significant role in its growth. Figure 1 briefly shows the sequence of the work in the present study.

## Materials and methods

### Microbial culture and maintenance

**Figure 2:**
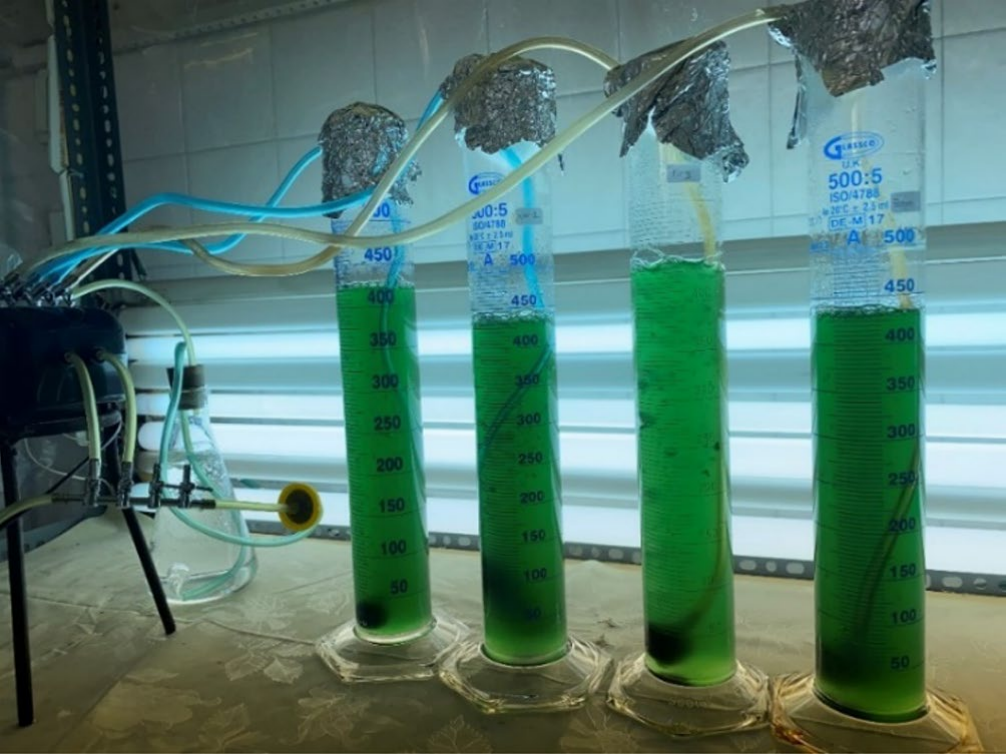
Inoculation of BG11 medium in graduated cylinder with *A. vaginicola*- the grown bacterium was used for the IIPBR study.

*A. vaginicola* isolated from rice (Oryza, sativa L.) was obtained from the previous study and all the necessary methods used in this study were in accordance with the relevant guidelines^13^. BG11 medium which contains synthetic nitrogen and carbon sources and other inorganic salts (NaNO_3_ and Na_2_CO_3_), was used to support growth of the test cyanobacterium^14^. Light is the driving force for photosynthesis and details of all these growth requirements and the cultivation process are given elsewhere^11,15^. Briefly, BG11 medium poured in several graduated cylinders, was inoculated with use of *A. vaginicola*. By installation of daylight fluorescent light lamps in horizontal position in front of graduated cylinder, the culture was illuminated, and a lux meter was used for measuring light intensity (LM 76 Light meter, Multimetrix^®^, China). The light at level of 80 µmol photons m^−2^ s^−1^ was provided. With use of laboratory tubing, CO_2_ was supplied through continuous aeration with filtered wet air (Fig. 2).

### Design, geometry, and operation of IIPBR

**Figure 3:**
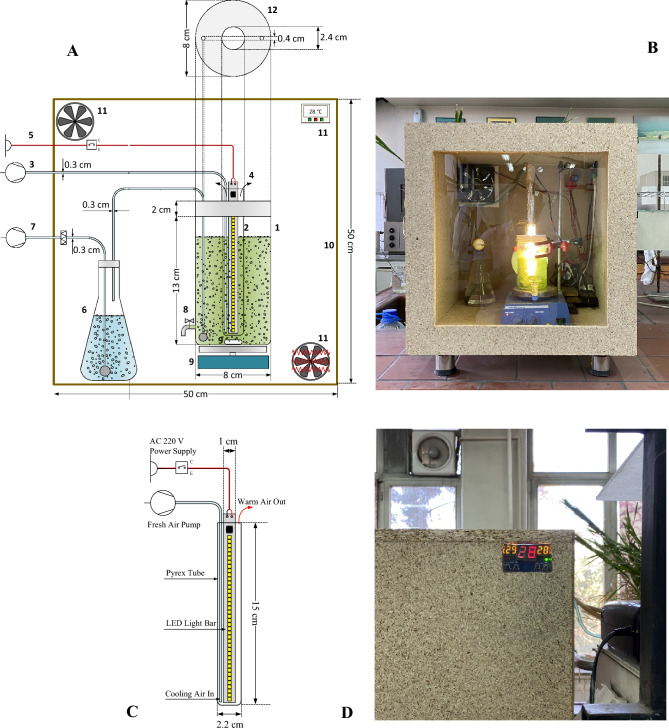
Schematic diagram showing experimental setup of the IIPBR schematically shown (**A**) see below for the details. The photograph of the photobioreactor which has been placed inside the incubator, is also presented (**B**). The dimension details of LED bar light which was placed in the Pyrex glass tube presented schematically (**C**). The photograph of temperature control switch sensor module is also given (**D**). (**A**) 1-main body of the IIPBR, 2-internal illumination system with use of the LED bar light positioned inside the Pyrex glass, 3-aquarium pump providing fresh air, 4-warm air outlet, 5-electrical power connection, 6-water container, 7-aquarium pump and relevant air filter, 8-sampling port, 9-magnetic stirrer and magnetic stir bar, 10-incubator, 11-system’s temperature controller, 12-top view of the IIPBR—See the text for the details.

Structural details of the experimental setup (600 ml as the nominal volume, 13 cm as height and 8 cm as the diameter) are presented in Fig. 3.

illumination details were as follows: Two 8 W LED bars (dimensions 30 × 1 cm) were used for the illumination where one bar was able to emit cool white light (6500 K) and the other one was capable to emit warm white light (3500 K), and both were connected to a 220 V AC power supply. Each of the LED bar consisted of 72 diodes with 180° beam angle (Tranyton Co. Ltd., Taiwan). LEDs were placed inside a Pyrex glass tube of 22 mm internal diameter. The temperature of an LED illumination system is higher than the ambient temperature (i.e., part of electric energy is converted to heat energy) and is advisable to use an appropriate system for controlling temperature. This adjustment simply was done by use of an aquarium pump (ACO-5505, HAILEA^®^, China) where fresh air would be available through connecting tubing (Fig. 3). For keeping temperature of the Pyrex glass surface at 28 °C, air flow rate was adjusted. Incubator made of chipboard (10 mm thickness), has the following dimensions (L* W* H): 50* 50* 50 cm. Electric coiling and fans were places at two positions and by fixing a temperature control switch sensor module at the top corner of the incubator (XH-W1401, Covvy^®^, China) each of these was able to respond appropriately to the temperature fluctuations. With all these considerations, the setup was finalized by placing the IIPBR inside the incubator and in this manner fluctuations of the system’s temperature were kept at the minimum level (± 1°C).

CO_2_ as the substrate was admitted into the IIPBR using an aquarium pump. Carbon dioxide was quantified in terms of aeration rate where air was passed through filter unit (0.22 μm) and water container (250 ml flask) (Fig. 3). Air flow rate was monitored by a flow meter (LZB-3WB, Changzhou Chengfeng^®^ Flowmeter, China). Wet air provides a favorable environment for growth and metabolic activity.

Table 1 presents the details of the experimental plans and the growth behavior was tested in terms of *A. vaginicola* expression to certain environmental stresses, i.e., Nitrogen content of the BG11 medium (0.5, 1.5 g/l as NaNO_3_ and the system without inorganic Nitrogen), quality and quantity of LED light bar (warm and cool white LED with intensity equaled to 80, 150, and 8 µmol photons m^−2^ s^−1^), and amount of CO_2_ expressed in terms of air flow rate (120, 40 ml/min, and system without aeration).

**Table 1:** Experimental plan used in the IIPBR considering different regimes of intensities of the LED light bar, inorganic nitrogen content of the growth medium, and amount of CO_2_ admitted to the reactor.

Treatment no.	Treatment variables		
	Nitrogen content of the BG11 medium as NaNO_3_ [g/l]	Intensity of LED light bar [µmol photons m^−2^ s^−1^]	Amount of CO_2_ admitted to the IIPBR [ml/min] (as air flow rate)
1	0.5	80	120
2	0.5	8	120
3	0.5	150	120
4	1.5	80	120
5	–^a^	80	120
6	0.5	80	40
7	0.5	80	–^a^

^a^Without the test variable.

*A. vaginicola* culture grown in graduated cylinder as described in Sect. 2.1, was used for the IIPBR study where the inoculum size was 4 ml/l considering 400 ml as the IIPBR working volume. IIPBR operation lasted for 11 days, and analyses were performed regularly (d^−1^) by taking appropriate sample in each time interval.

### Analytical methods

The data were presented properly, using one-way analysis of variance (ANOVA test- Microsoft Excel 2021). The level of significance was set at α = 0.05 (i.e., type I error- accepting the alternate hypothesis when the null hypothesis is true) and 95% of the reported value lies between ± 3 SD (standard deviation).

Growth of the cyanobacterium was determined spectrophotometrically in the rage of 380 to 800 nm (V-550 UV/VIS Spectrophotometer, JASCO^®^, Italy) where the absorption peaks were used for quantification. Gravimetric method was used to measure biomass dry weight, i.e., the cells were collected daily using 10 ml of sample and by Büchner funnel liquid portion was separated and the wet residue remained on the funnel surface (Whatman filter paper 4) was dried in a laboratory oven (105°C for 24 h). The data fitting was performed using regression analysis.

A further note was to extract lipids from the test cyanobacteria and the obtained content was estimated^15,16^. Different amounts of chloroform and methanol were used at the dried biomass and homogenization and filtration were carried on according to the details given in the relevant reference. The Chloroform solvent was let to evaporate, and the residue was used in the gravimetric methods and the lipids content was determined.

### Growth kinetics and its relationship to lipids production

A typical growth curve for *A. vaginicola* was obtained using exponential growth model:

1 $${C}_{t}={C}_{i}{e}^{\mu .t}$$

where C_i_ is the biomass content (g L^−1^) at initial stage, C_t_ is biomass at any time t during experiment, and µ is the growth rate constant (d^−1^). For measuring the µ, the linearized form the Eq. 1 was used:

2 $$\mu =\left(ln {C}_{t}-\mathit{ln}{C}_{0}\right)/t$$

With use of the growth rate constant, biomass productivity was determined (mg L^−1^ d^−1^):

3 $${P}_{B}={C}_{t}\times \mu $$

Capacity of *A. vaginicola* for production of lipids was determined in terms of lipids yield (%):

4 $${Y}_{L}=\left({W}_{L}/{W}_{d}\right)*100$$

where *W*_*L*_ is the weight of the total lipids (g), and *W*_d_ is the weight of the dry biomass (g).

Further approach on *A. vaginicola* was to measure lipids productivity (mg L^−1^ d^−1^) based on the following equation:

5 $${P}_{L}=\left({P}_{B}*{Y}_{L}\right) / 100$$

### IIPBR energetics

Considering different forms of energies which participate in functionality and operation of these types of photobioreactors, is a reasonable approach for giving an estimate for the process cost as described in the results and discussion section. The point of interest in the present study is IIPBR’s energy utilization in terms of energy of the LED light bar and energy consumed for mixing operation as the input energy. Biomass productivity and the lipids formation by the *A. vaginicola* cyanobacterium culture under selected operation variables described in the experimental (Table 1) were measured.

On the bases of energy required for system’s illumination (E_L-_ W m^−3^), IIPBR performance was assessed, and comparison was made with other types of the photobioreactors (PBRs). E_L_ was estimated in terms of power input per unit culture volume^17^:

6 $${E}_{L}=\frac{0.22{I}_{o}A}{V}$$

where I_o_ is incident light intensity per unit incident area (µmol m^−2^ s^−1^), A is incident area (m^2^), and V is the culture volume (m^3^).

Similar approach was used for estimation of mixing energy input per unit culture volume (E_M,B_-W m^−3^)^18^:

7 $${E}_{M,B}=\frac{Q\gamma h}{60V}$$

where, Q is the volumetric gas flow rate (m^3^ min^−1^), γ is the specific weight of the broth (N m^−3^), h is the culture depth (m), and V is culture volume (m^3^).

On bases of total energy used for IIPBR performance, biomass productivity per unit power input (P_UV_-g W^−1^ d^−1^) was estimated and used for further comparisons with other photobioreactors^19^:

8 $${P}_{UV}={P}_{B}/({E}_{L}+{E}_{M,B})$$

## Results and discussion

Photosystem (PS)I, PSII, and the associated phycobilisomes (PBSs) (which contain phycobilins as chromophores among different molecular species), are major photosynthetic pigments in cyanobacteria. Cooperative action of PSI and PSII upon receiving light (quality of light characterized by its wavelength) leads to charge separation process in which O_2_ as strong oxidant is formed and movement of the excited electron through the formed electron carrier results in formation of strong reductant (NADPH). While the hydrogen ion accumulation across the thylakoid membrane is calculable in terms of pH gradient which acts as driving force for enzymatic synthesis of ATP from ADP and P_I_. The occurrence of all these events is for a system being illuminated under ordinary/natural growth condition. Interpretation of the results depends on the environmental condition used for cyanobacterium cultivation. In fact, not all genes of an organism express during ordinary growth condition and expressiveness of some genes depend on environmental stress being sensed by the cell where the response of the cell with theses received signals (chemical/physical) controls the organism performance. i.e., the cell tries to find a proper strategy to cope with this imposed condition. Histidine protein kinases (HPKs) are a diverse group of signal transduction enzymes which their catalytic role is in the transfer of phosphate group from molecules having high phosphate group potential (such as ATP) to a histidine residue^20^. Several genes encode HPKs have been isolated in cyanobacteria where the gene assigned to HK2 is found in all cyanobacteria and functionality of these protein is like chloroplast sensor kinase (CSK) and regulatory role of CSK is in redux state in plants and algae during changes in light quality^20,21^. HPKs are also associated in thylakoid membrane (TM) where this membrane is the site for production of most ATP, NADPH, and the carrier molecules in chains of electron transport in photosynthesis and in respiration^22^.

Inorganic nitrogen of BG11 growth medium, light substrate (quality and quantity), and CO_2_ substrate (quantified in terms of aeration rate) were the variables studied in the present work and the findings have been discussed with reference to Histidine kinases being expressed under ordinary and stressful conditions.

### Light as the substrate

**Figure 4:**
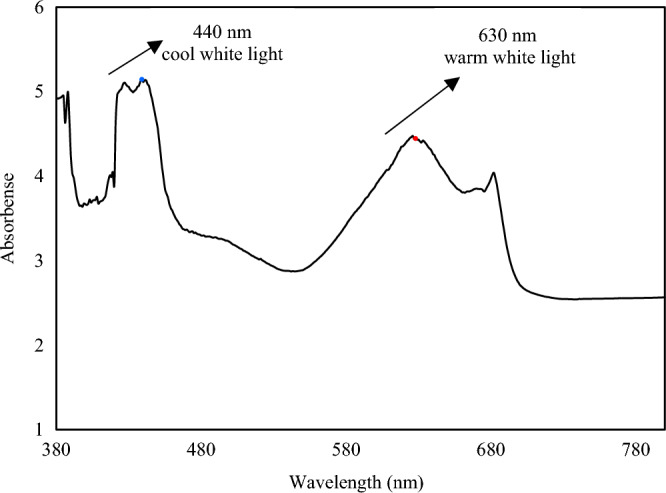
Specification of spectrum of cool and warm white LED lights in the IIPBR used to monitor *A. vaginicola* growth.

Autotrophic condition used to monitor the cyanobacterium *A. vaginicola* growth in IIPBR which was under continuous mode of light illumination, was practiced in the present study. cool and warm white LED light having narrow spectral ranges of 400–450 and 580–650 nm were the source of system’s illumination and Fig. 4 shows *A. vaginicola* growth under this specified condition.

Study on cyanobacterium *Synechocystis* sp. PCC6803 showed that the bacterium absorbed blue light to a similar extent as orange and red light, but it was less able to use it effectively in oxygenic photosynthesis^23^. The results supported the hypothesis that blue light could create an imbalance between the two PSs where excess energy found to be associated with PSI side which contained more chlorophyll ‘a’ than PSII and the PSII side was less efficient and O_2_ production was not effectively proceeded. PSII association with Phycobilisomes (PBSs) increases chance of the PSII participation in synthesis of ATP. It was also found that when intensity of the blue light was high enough to saturate PSII, the O_2_ production rate in the blue light was close to the rate found in orange and red light^23^. further, one should consider performance of beta-carotene (absorbing blue and green wavelength) more abundant in PSI than PSII and this also contribute photosynthetic light harvesting antenna on PSI.

The findings of the present work agree with the results reported on effects of the LED light (combined forms of blue and red LED light) on *Nannochloropsis oculuta* and *Tetraselmis chuii*^24^.

**Figure 5:**
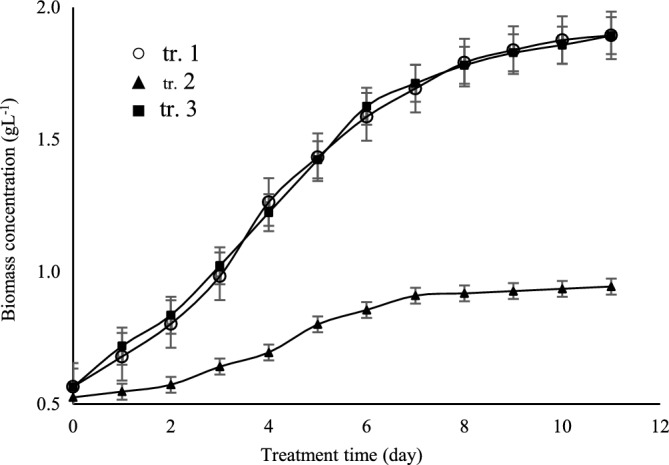
Growth curve of *A. vaginicola* cultured under influence of light intensity as described in Table 2.

Table 2 shows that the 8 µmol photons m^−2^ s^−1^ did not support *A. vaginicola* growth and growth rate constant was 45% lower than the other two test intensities values where biomass productivity, lipids yield, and lipids productivity were close for 80 and 150 µmol photons m^−2^ s^−1^ where 80 µmol photons m^−2^ s^−1^ was chosen for further experiments mainly because of operational costs **(**Fig. 5**).** However, the lipids yield in terms of percentage was similar for all three test intensities (Table 2). The regulatory role of genes of HPKs in completion of heterocystous structure is also interesting in nitrogen fixation ability of these cells. Synthesis of glycolipids at the end of growth stage facilitates differentiation of heterocyst from vegetative cells where resistance of the envelope layer composed of the glycolipids, is enough to prevent entry of oxygen into the heterocyst^25^. This study indicates the decisive role of lipids in heterocyst structure, thus nitrogen fixation ability of the filamentous cyanobacteria.

**Table 2:** *A. vaginicola* performance under influence of light intensity.

Treatment no.	Light intensity [µmol photons m^−2^ s^−1^]	Specific growth rate [d^−1^]	Biomass productivity [mg L^−1^ d^−1^]	Lipids yield [%]	Lipids productivity [mg L^−1^ d^−1^]
1	80	0.11 ± 0.03	202.60 ± 9.27	7.63 ± 2.11	15.65 ± 4.98
2	8	0.05 ± 0.01	50.31 ± 2.63	7.71 ± 1.47	3.92 ± 0.94
3	150	0.11 ± 0.02	198.43 ± 6.74	7.61 ± 0.98	15.17 ± 2.46

### Inorganic nitrogen as the substrate

With use of exponential equation *A. vaginicola* growth was modeled and the effect of inorganic nitrogen (NaNO_3_) as the main constituent of BG11 medium was examined. Table 3 shows the cyanobacterium performance under influence of nitrogen at three different concentrations which were considered in the present study when illumination of IIPBR was set at 80 µmol photons m^−2^ s^−1^ LED intensity.

**Table 3:** *A. vaginicola* performance under influence of nitrogen content of the BG11 medium (as NaNO_3_) illumination of IIPBR was set at 80 µmol photons m^−2^ sn^1^ LED intensity.

Treatment no.	Nitrogen content [g/l]	Specific growth rate [d^−1^]	Biomass productivity [mg L^−1^ d^−1^]	Lipids yield [%]	Lipids productivity [mg L^−1^ d^−1^]
1	0.5	0.11 ± 0.03	202.60 ± 9.27	7.63 ± 2.11	15.65 ± 4.98
4	1.5^a^	0.06 ± 0.01	62.26 ± 4.34	6.52 ± 3.14	4.20 ± 2.24
5	No NaNO_3_	0.11 ± 0.04	193.10 ± 8.65	7.54 ± 1.96	14.73 ± 4.44

^a^Considering as BG11_std_.

**Figure 6:**
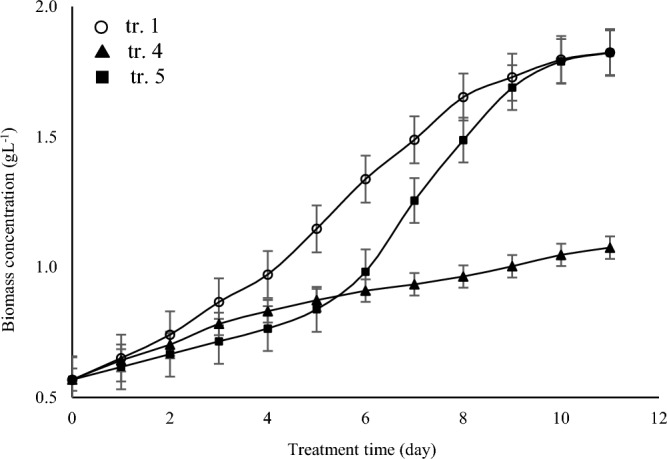
Growth curve of *A. vaginicola* in culture medium influence of nitrogen content of the BG11 medium (as NaNO_3_) as describe in Table 3.

Quaintly and quality of carbon and nitrogen sources being used for culturing cyanobacteria are sensed by the bacterium and how the cells respond to the flow rates of theses bioelements and how the cells metabolically gain ability to maintain balanced situation for utilization of these elements. Study on glutamine synthetase (GS) for instance shows importance of the enzyme in catalytic conversion of inorganic nitrogen to amino acid (reductive amination reaction)^1^. As has mentioned above, studies focusing on signaling mechanism have indicated crucial roles of environmental stresses on cyanobacteria performance (two-component system…)^21,22,26^. Findings in Table 3 show that *A. vaginicola* cultivation in a medium containing 1/3 of NaNO_3_ present in BG11 medium, gave a three-fold increase in lipids productivity and similar increase was observed for the biomass productivity. Figure 6 shows that growth of *A. vaginicola* in BG11 which prepared without NaNO_3_ was gradually increased up to 6^th^ day of the cultivation in IIPBR and thereafter it was raised and interestingly the measured $\mu$ value was remarkably close to the test cyanobacterium cultured in BG11 prepared with 2/3 decrease in NaNO_3_ (Table 3).

Study on cyanobacteria showed that the bacteria were able to accumulate cyanophycin (a nitrogen-rich polypeptide as the light storage material) under nitrogen-poor conditions and this appears to be a strategy used by cyanobacteria through optimized nitrogen assimilation^27^. Acyl carrier protein, acetyl CoA carboxylate, … all are proteins involved in lipids biosynthesis pathway^1^.

A reasonable approach in describing these types of results is to consider involvement of different strains of Anabaena Sp., formation of (some) intermediates acting as a signal molecule due to activation of (some) genes related to NtcA-light system. The importance of the signaling mechanism and its applicability should be appreciated in any future work.

### Effect of CO_2_ expressed in terms of aeration flow rate

The rate limiting step in carbon fixation, is addition of the gaseous form of the inorganic carbon to the phosphorylated ketose catalyzed ribulose 1, 5-bisphosphate carboxylase-oxygenase (rubisco) where its half saturation constant in cyanobacteria is in the range of 100–180 micromolar and this reported finding in the literature is despite of low atmospheric concentration of CO_2_. Carbon dioxide concentrations mechanism (CCMs) developed in cyanobacteria consists of several steps being expressed as a plan used by the cells and these abilities to effectively increase the CO_2_ concentration around rubisco active site and this decrease’s chance of rubisco to catalyze oxygenation (photorespiration). First consideration in CCMs is passage of the hydrated form of CO_2_ (HCO_3_^−^) from the cell membrane through the synthesized transporters, diffusion of hydrogen carbonate across the shell of carboxysomes as protein micro compartment where carbonic anhydrase catalysis conversion of HCO_3_^−^ to CO_2_^28,29^.

**Figure 7:**
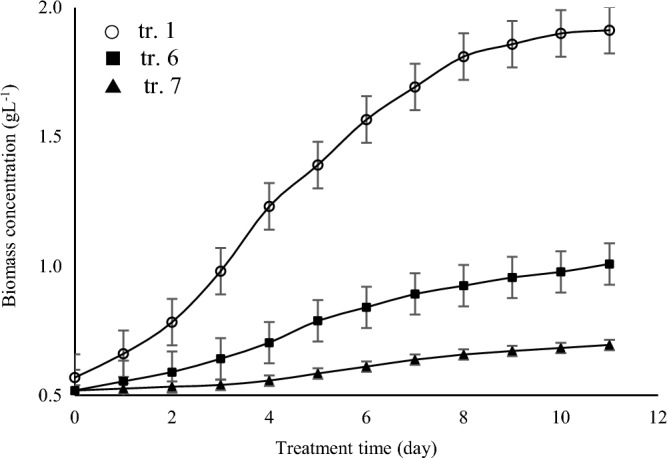
Growth curve of *A. vaginicola* in culture medium under influence of CO_2_ (expressed in terms of aeration flow rate) as described in Table 4.

Table 4 shows *A. vaginicola* performance under influence of CO_2_ (expressed in terms of aeration flow rate) when the nitrogen concentration used in the experiment was 0.5 g L^−1^ and the IIPBR was illuminated at 80 µmol photons m^−2^ s^−1^ When highest aeration rate used in the present study (120 ml/min) *A. vaginicola* relied on its CCMs plan which corresponded to growth quality maintenance. A significant increase in biomass productivity was obtained compared to the conditions of cells culturing under no aeration **(**Fig. 7**)**. Decrease of the aeration to 1/3 did not have effect on the lipids yield this condition affected negatively on production of biomass and lipids.

**Table 4:** *A. vaginicola* performance under influence of CO_2_ (expressed in terms of aeration flow rate) when the nitrogen concentration used in the experiment was 0.5 g L^−1^ and the IIPBR was illuminated at 80 µmol photons m^−2^ s^−1^.

Treatment no.	CO_2_ expressed in terms of air flow rate [ml/min]	Specific growth rate [d^−1^]	Biomass productivity [mg L^−1^ d^−1^]	Lipids yield [%]	Lipids productivity [mg L^−1^ d^−1^]
1	120	0.11 ± 0.03	202.60 ± 9.27	7.63 ± 2.11	15.65 ± 4.98
6	40	0.06 ± 0.01	61.92 ± 8.28	7.06 ± 2.18	4.55 ± 1.93
7	No aeration	0.03 ± 0.01	18.48 ± 1.79	6.83 ± 0.98	1.28 ± 0.30

### Literature survey and data comparison

The data of the present study shown in Table 5 has been used to compare performance of several designs of PBRs, focusing on light energy (W m^−3^), and mechanical energy (W m^−3^) as related biomass production (g l^−1^ d^−1^) based on the involved microorganisms. PBR efficiency depends on the performance of the microorganism in capturing light energy and its utilization where the system needs to maintain relatively constant production of biomass. Findings reported by Zittelli et al.^35^ show that biomass productivity in 140 L PBR was close to the value obtained in the present study (Table 5). The small working volume test PBR allows ease of conducting research about cyanobacterial growth under varying conditions with or without imposing stressful environment. Where the microbe’s responses can be analyzed and examined in more detailed experiments. The biomass productivity value reported in terms of light and mechanical energy in the present study shows better performance of the system suggested in comparison with other PBRs listed in Table 5.

**Table 5:** Characteristics of photobioreactors used in different studies.

Type of PBR^a^	Micro-organism^b^	Working volume (Lit)	Growth media	Gas: liquid ratio (m^3^ min^−1^ m^3^)	Light			Reactor’s input energy		Biomass productivity		References
					Incident area (m^2^)	Intensity (µmol m^−2^ s^−1^)	Type ^c)^	Light energy (W m^−3^)	Mechanical energy (W m^−3^)	P_B_ (g l^−1^ d^−1^)	P_UV_ (g W^−1^ d^−1^)	
TRC	*Ch*	18	Walne’s medium	0.30	0.05	660	H	399.1	9.7	0.30	0.73	^30^
BC	*Ap*	3	BGN medium	1.0	0.18	150	F	1923.5	122.5	0.77	0.38	^31^
BC	*Ch*	0.6	Allen medium	0.2	0.03	100	F	1212.5	15.1	0.34	0.27	^32^
BC	Ch*	0.8	modified *f/2* medium	0.25	0.07	300	F	5385.9	8.5	0.50	0.09	^33^
BC	*No*	0.8	modified *f/2* medium	0.25	0.07	300	F	5385.9	8.5	0.42	0.08	^34^
A	*Na*	120	*f* medium	0.1	9.3	89	F	1315.3	23.3	0.20	0.15	^35^
A	*Na*	140	*f* medium	0.1	9.3	133	M	1965.5	23.3	0.25	0.13	^35^
MFPP	*Na*	123	modified *f/2* medium	0.5	3.40	230	F	8304.5	129.0	0.97	0.12	^36^
Helical	*Sp*	8	Medium for *Spirulina*	0.038	0.65	197	F	3496.7	5.3	0.51	0.15	^37^
IIPBR	*Sc*	18	Bold’s basal medium	0.044	0.25	91.4	F	276.7	5.9	0.40	1.42	^19^
IIPBR	*Ns*	18	Bold’s basal medium	0.044	0.25	91.4	F	276.7	5.9	0.1	0.34	^19^
IIPBR	*Av*	0.5	BG-11	0.24	0.008	80	LED	281.6	4.2	0.21	0.74	Present study
EIPBR	*Av*	0.5	BG-11	0.24	0.015	80	F	528	4.2	0.21	0.39	Present study

^a^TRC transparent rectangular chamber; BC bubble column; FPA flat panel airlift; A annular; MFPP modular flat plate panel, EIPBR externally illuminated photobioreactor; ^b^*Ch*, *Chlorella*; Ap, Aphanothece microscopic Nageli; Ch*, Chlorella sp.; *No*, *Nannochloropsis oculta*; *Sp, Spirulina platensis*; *Na, Nannochloropsis*; *Sp, Spirulina*; *Sc*, *Scenedesmus* sp.; *Ns, Nannochloropsis salina; Av, Anabaena Vaginicola*; ^c^*H* halogen lamp; *F* florescent lights; *M* metal halide lights.

The findings of the present study on A. *vaginicola* behavior in the IIPBR which performed under different levels of CO_2_ and light intensity are beneficiary by conducting a soil experiment by interested researcher(s) where one uses A. *vaginicola* as the soil inoculant, selects a model plant, and records changes occur in characteristics of the tested soil and the grown plant. This approach has been used by Kokila et al., ^38^ who used each of A. *laxa* and A. *torulosa* as the soil inoculant and monitor growth of tomato as the model plant.

Recent report on nitrogen fixing cyanobacteria as a potential resource for biodiesel production, better indicates complex nature of these prokaryotes where decision making process based on the growth rate constant, lipid productivity, etc. is not an easy task^39^. Table 6 prepared with use of the data presented in that report shows the extent of the data wideness. Cyanobacterium *A. vaginicola* occupies an acceptable position. Recognition of gene-based strategy followed by cyanobacteria is important and may help to explain the behavior of these wonderful microorganism in responding to environmental changes.

**Table 6:** Growth behavior for some nitrogen fixing cyanobacteria^39^.

Species	growth rate constant [d^−1^]	Biomass productivity [mg L^−1^ d^−1^]	Lipids yield [%]	Lipids productivity [mg L^−1^ d^−1^]
*Sysnechosis sp.*	0.31			15.27
*Anabaena cylindrica*	0.27 ± 0.05	303.06 ± 41.6	6.95 ± 0.2	21.02 ± 2.3
*Anabaena cycadeae*	0.27 ± 0.03	131.67 ± 2.46	9.75 ± 0.25	12.84 ± 0.58
*Anabaena vaginicola*	0.11 ± 0.03	202.60 ± 9.27	7.63 ± 2.11	15.65 ± 4.98

The design of PBR internally illuminated with LED light bar in the present study was adequate and supported cyanobacterium *A. vaginicola* growth under nitrogen limited condition (correlation with lipids production). The design of IIPBR reported by Pegallapati et al. appeared to be good enough for growth of *Scenedesmus* sp.^19^. It is reasonable to see different approaches used for comparison between these microorganisms (Table 5).

With use of fluorescent lamp instead of LED bars and placed four of them outside the reactor (20 cm distance) in the present study, the biomass production by *A. vaginicola* cultivation under externally illuminated process was practiced (same operational conditions as IIPBR for *A. vaginicola* growth, introduced as EIPBR in Table 5). By this experimental work, comparison of the IIPBR with its externally illuminated counterpart, appears to make comparison more reasonable where the P_uv_ was 48% higher in the IIPBR (0.74 vs 0.39 g w^−1^ d^−1^). The presence of the novel air-cooled system in the reactor has placed IIPBR economically in favorable position.

## Conclusions

*Anabaena vaginicola* is the genus of filamentous cyanobacteria with nitrogen fixing abilities. Extent of lipid production of *A. vaginicola* as was recently identified, evaluated through its cultivation in the IIPBR designed in the present study. Light intensity, CO_2_ level, and inorganic nitrogen content of the growth medium as environmental factors which were undertaken by test microorganism, managed, and controlled efficiently (i.e., 80 µmol photons m^−2^ s^−1^ as the light intensity, 0.5 g L^−1^ as the inorganic nitrogen source, and 120 ml min^−1^ as aeration rate as the CO_2_ substrate). The findings were discussed based on the two component systems having regulatory function and the expressiveness is well correlated with the environmental changes sensed by cyanobacteria. Calculated IIPBR energetics as the value of biomass productivity per unit power input has placed the IIPBR in favorable position compared to other photobioreactors as discussed in the above text.

The results are extendible by conducting a soil experiment by interested researcher(s). Use of grown *A. vaginicola* as soil inoculant is advantageous to poor soils.

### Supplementary Information

Supplementary Information 1.

Supplementary Information 2.

Supplementary Information 3.

Supplementary Information 4.

## Data Availability

All data generated or analyzed during this study are included in this published article and its supplementary information files 1, 2, 3, and 4.
