# Supplementary material for: Lipids productivity of cyanobacterium Anabaena vaginicola in an internally illuminated photobioreactor using LED bar lights
Source: Sci Rep. 2024 Mar 21;14:6857. doi: 10.1038/s41598-024-54414-0 (PMC10957962; doi:10.1038/s41598-024-54414-0)
Supplement: Supplementary file 1 — Supplementary Information 1. [file 41598_2024_54414_MOESM1_ESM.docx]

**Supplementary File: Raw Data**

Lipids Productivity of Cyanobacterium *Anabaena vaginicola* in an Internally Illuminated Photobioreactor Using LED Bar Lights

1. **Raw Data Gathered from Spectrophotometer**

Datasets used in this study include all received data from Spectrophotometer (V-550 UV/VIS, JASCO®, Italy).

For each of three variables considered in this study (i.e., Illumination, Nitrogen Source, and Aeration), three scenarios have been considered as described in Table 1. of the manuscript.

As per the experimental plan described in manuscript, for each treatment, daily samples were collected, and their Optical Density was calculated via specified spectrophotometer above.

Attachments 1, 2 and 3 of the supplementary file include all collected data from spectrophotometer for Illumination, Nitrogen Source, and Aeration variables, respectively (as Excel files).

In each attachment, there is a “Spectro Results” tab which represents graphical results of collected data from spectrophotometer (as growth curve) including considered operation conditions for each scenario.

Calculations resulted from the gathered data have been presented in “Growth” tab of each attachment. In the tab file:

1. Optical densities of two observed peaks (i.e., 440 and 630 nm) in the growth curves for each day were gathered.
2. As per gathered data, µ was calculated as per equation 2 specified in the manuscript.
3. Also, biomass productivity for each scenario was calculated as per equation 3 specified in the manuscript. Results of these calculation (along with lipids yield and lipids productivity) have been presented in tables 2 to 4 of the manuscript.
4. Considering the method specified in clause 2.3 of the manuscript (Analytical Methods), biomass dry weight as mass density (g/l) was calculated for each scenario which the results have been presented in “Growth” tab of each attachment.
5. An accumulative graph of biomass density vs day for all scenarios of each variable has been drawn in the specified tab for comparison and analysis of the results. Specified graphs have been presented in Figures 4 to 6 of the manuscript along with their deep analysis.

1. **Data required for the IIPBR setup**

**Data availability statement:** All data required for IIPBR setup during are included in this published article. For details, please check clause 2.2 of the manuscript.
